# Is the Critical Care Resuscitation Unit Sustainable: A 5-Year Experience of a Beneficial and Novel Model

**DOI:** 10.1155/2022/6171598

**Published:** 2022-07-19

**Authors:** Elizabeth Powell, Iana Sahadzic, Daniel Najafali, Emilie Berman, Katie Andersen, Leenah Z. Afridi, Zoe Gasparotti, Erin Niles, Jeffrey Rea, Thomas Scalea, Daniel J. Haase, Quincy K. Tran

**Affiliations:** ^1^The R Adams Cowley Shock Trauma Center, University of Maryland School of Medicine, Baltimore, MD, USA; ^2^The Research Associate Program in Emergency Medicine and Critical Care, Department of Emergency Medicine, University of Maryland School of Medicine, Baltimore, MD, USA; ^3^Carle Illinois College of Medicine, University of Illinois Urbana-Champaign, Champaign, IL, USA; ^4^University of Maryland School of Medicine, Baltimore, MD, USA; ^5^Department of Emergency Medicine, University of Maryland School of Medicine, Baltimore, MD, USA

## Abstract

**Background:**

The 6-bed critical care resuscitation unit (CCRU) is a unique and specialized intensive care unit (ICU) that streamlines the interhospital transfer (IHT—transfer between different hospitals) process for a wide range of patients with critical illness or time-sensitive disease. Previous studies showed the unit successfully increased the number of ICU admissions while reducing the time of transfer in the first year of its establishment. However, its sustainability is unknown.

**Methods:**

This was a descriptive retrospective analysis of adult, non-trauma patients who were transferred to an 800-bed quaternary medical center. Patients transferred to our medical center between January 1, 2014 and December 31, 2018 were eligible. We used interrupted time series (ITS) and descriptive analyses to describe the trend and compare the transfer process between patients who were transferred to the CCRU versus those transferred to other adult inpatient units.

**Results:**

From 2014 to 2018, 50,599 patients were transferred to our medical center; 31,582 (62%) were non-trauma adults. Compared with the year prior to the opening of the CCRU, ITS showed a significant increase in IHT after the establishment of the CCRU. The CCRU received a total of 7,788 (25%) IHTs during this period or approximately 20% of total transfers per year. Most transfers (41%) occurred via ground. Median and interquartile range [IQR] of transfer times to other ICUs (156 [65–1027] minutes) were longer than the CCRU (46 [22–139] minutes, *P* < 0.001). For the CCRU, the most common accepting services were cardiac surgery (16%), neurosurgery (11%), and emergency general surgery (10%).

**Conclusions:**

The CCRU increases the overall number of transfers to our institution, improves patient access to specialty care while decreasing transfer time, and continues to be a sustainable model over time. Additional research is needed to determine if transferring patients to the CCRU would continue to improve patients' outcomes and hospital revenue.

## 1. Introduction

Critically ill patients with a wide range of complex disease processes often require expeditious transfer to tertiary or quaternary institutions' emergency departments (EDs) and intensive care units (ICUs) for a higher level of care. Decreasing time to definitive care at high-volume centers can improve outcomes in various patient populations, and it is for this reason that in July 2013, the critical care resuscitation unit (CCRU) was established at the University of Maryland Medical Center (UMMC)'s R Adams Cowley Shock Trauma Center (STC) [1–5]. The CCRU was designed to be a multispecialty resuscitation unit that receives both medical and surgical patients, typically from Maryland, Delaware, and Virginia. The unit's mission is to receive patients with time-sensitive critical illness, provide stabilization, and transfer them to definitive care or the destination traditional intensive care unit (ICU), typically within 6 to 12 hours. The resources, training requirements, and staffing of the CCRU differs from those of an ED. Unlike the ED, the nurse-to-patient ratio of the CCRU is strictly maintained at 1 : 1 or 1 : 2, and nursing staff need at least 2 years of critical care nursing experience to staff the CCRU. The unit has 6 beds and is staffed by attending intensivists 24 hours a day as well as critical care advanced practice providers and critical care nurses trained in a broad spectrum of medical and surgical critical care. While originally designed to receive patients from other hospitals, the CCRU also admits patients who clinically deteriorate while being treated within the UMMC/STC institution when a bed is not available in other traditional ICUs. The CCRU is an ICU-based resuscitation unit; therefore, the CCRU can accept transfers from other ICUs or provide an ICU bed when patients at our institution deteriorate and need an immediate ICU bed.

Prior to the CCRU establishment, our institution experienced growth in cardiac surgery, extracorporeal membrane oxygenation, vascular surgery, neurosurgery, stroke, and acute care emergency surgical services programs. However, due to a lack of ICU bed availability, up to 25% of appropriate candidates could not be transferred [6]. After the establishment of the CCRU, critically ill patient volume increased while transfer time decreased [6]. Time intervals from referring ED transfer to ICU arrival along with associated mortality also decreased when patients were transferred to the CCRU as opposed to the traditional ICUs [7]. Patients with large vessel occlusions arrived to the CCRU faster than the subspecialty neurocritical care unit (NCCU) and had similar outcomes [8]. Similarly, patients with spontaneous intracranial hemorrhage requiring neurosurgical intervention arrived to the CCRU in a shorter amount of time than the NCCU and had external ventricular drains (EVDs) placed in a similar timeframe while receiving continuing care and blood pressure control [9, 10]. These studies demonstrate that the CCRU can more rapidly facilitate the transfer of patients with critical, time-dependent illness and provide a high level of subspecialty care, similar to those provided by our subspecialty ICUs.

However, there remained the question about the increased rate at which patients are brought to our quaternary medical center via the CCRU and the ability of the CCRU to continue to be a sustainable model. If there are more patients being admitted to our medical center with a finite number of available beds, will this increase eventually exceed the number of available beds? This could potentially create a backlog for patient transfer requests and the process of transfer delay will eventually repeat itself. This information will be essential for administrators and researchers at other institutions to consider when they plan to establish a similar resuscitation unit at their own institutions. Therefore, our goal is to describe the volume of patients transferred to the CCRU and its contribution to the operations of UMMC/STC transfers over a longitudinal period. Our primary aim is to assess if ICU transfer volume has changed through the utilization of the CCRU over a 5-year period from 2014 to 2018. Our secondary aim is to assess the time intervals for patients who were transferred to the CCRU over the same longitudinal period of time. Sustainability is shown through the capability of the CCRU to maintain the number of admissions, improve bed assignment for patients, and keep the number of lost admissions low.

## 2. Methods

### 2.1. Study Design and Clinical Settings

This is a descriptive, retrospective analysis of transfer records of patients who were transferred from another hospital to the University of Maryland Medical Center (UMMC), which is an 800-bed quaternary medical center. The UMMC has 5 adult non-trauma specialized ICUs: (1) cardiac surgical ICU (CSICU), (2) coronary care unit (CCU), (3) neurocritical care unit (NCCU), (4) medical ICU (MICU), and (5) surgical ICU (SICU). The study period was January 1, 2014 to December 31, 2018, as the CCRU's opening at the end of July 2013 makes the year 2014 the first full calendar year for the operation of the CCRU. With the exception of the CCRU's opening, there were no major capacity changes (e.g., additional ICU beds added, operating room changes, or capacity increases) to our medical center during this time period. Our study met exemption status by our Institutional Review Board (IRB) for formal consent.

### 2.2. Patient Selection

Adult, non-trauma patients who were transferred to our medical center between June 1, 2012 to December 31, 2018 were eligible. We compared a convenient sample of patients who were transferred to UMMC between January 1, 2014 and December 31, 2018 for a total of five calendar years. Patients transferred to the CCRU during this period were compared with patients who were transferred to other inpatient units (ICU or medical or surgical wards) or to the emergency department (ED) at our medical center. Additionally, the number of patients transferred from June 1, 2012 to June 30, 2014 were used as the historical data for our interrupted time series (described in greater detail in the Statistical Analysis subsection), which investigated the trend of transfers before and after the creation of the CCRU. We did not include trauma patients because our medical center admits interhospital-transferred trauma patients directly to our regional stand-alone trauma center—the Trauma Resuscitation Unit (TRU). Critically ill trauma patients from the TRU are then admitted to two specialized trauma ICUs (neurotrauma ICU and multi-trauma ICU), rather than the adult non-trauma ICUs at our medical center. We excluded patients initially accepted for transfer to UMMC, but later transferred to another hospital (lost admissions), as we would not have any transfer information for these patients. We did however include the number of lost admissions over the course of the 5-year period to complete the transfer picture at the CCRU and our institution. We also excluded patients whose transfer data were missing.

### 2.3. The Transfer Process

All transfer requests for patients from another hospital to the UMMC are handled by our institution's transfer center (Maryland ExpressCare, MEC). If the referred patient has a critical illness requiring ICU admission, or they have a time-sensitive disease requiring urgent intervention, MEC will connect the sending clinicians with the specialty physicians and the physicians in charge of the bed allocation in the receiving ICU at UMMC.

When the patients are considered appropriate for transfer and the respective specialty ICU does not have an immediate bed available, the CCRU attending physician is included in the consult. It is during this conversation that the CCRU physician will consult with the specialty physician regarding a care plan and subsequently assign a bed for the patient according to the urgency of their needs. Patients who have high acuity or time-sensitive disease requiring urgent interventions typically receive high priority for a CCRU bed assignment. It is the responsibility of the sending physician to decide the type of transport (air vs. ground) or the level of transport (e.g., nurse vs. paramedic) according to the patient's acuity and the availability of transport teams. We measured transfer request to bed assignment time in minutes. Transfer time for this calculation was considered from the time the physicians at the sending hospitals contacted the CCRU physicians and the specialty physicians at our hospital to request transfer to the time the patient was assigned a bed in the CCRU. Therefore, time zero would be the time the outside hospital contacted the CCRU to transfer their patient.

### 2.4. Data Collection

Transfer records for patients who were transferred to the UMMC were collected and maintained by MEC as part of the transfer center's clinical operations. From the MEC database, we identified the transfer request time, bed assignment time, and patient arrival time at the UMMC. We identified the destination units that the patients were to be transferred to as well as the specialty service (i.e., acute care surgery, neurosurgery, etc) that accepted the patients and cared for them during their hospitalization at our medical center.

### 2.5. Outcome Measures

Our primary outcome was the volume of patients who were transferred to the CCRU in each year of the 5 years since its opening. Our secondary outcome was the time interval from transfer request to assignment of an available bed at the CCRU or other inpatient units at our medical center.

### 2.6. Statistical Analysis

When reporting our descriptive statistics, we used frequencies (percent, %) for categorical variables and mean (±standard deviation, SD) or median (interquartile range, IQR) for continuous variables, as appropriate. Student's *t*-test was used to compare the means, while Mann–Whitney *U* test was used to compare medians between groups. We used Pearson's chi-squared test or Fisher's exact test to compare categorical data based on the respective frequencies of the endpoint in question.

To examine the effect of the CCRU establishment on the trend of the number of ICU patients being transferred to the UMMC before and after the opening of the CCRU, an interrupted time-series analysis (ITS) was performed. The ITS examines the effect of an intervention on a population by comparing the trend before and after an intervention. For our ITS, we defined the intervention period as July 2013, the year the CCRU was established. Therefore, the ITS was constructed with a historical number of patients who were transferred to any adult non-trauma ICU during each month between June 1, 2012 until June 30, 2013 versus the monthly number of ICU patients who were transferred to UMMC between January 1, 2014 and December 31, 2018.

The ITS analysis was performed in Python (version 3.7.1 with *Pandas* 1.1.0 package). Other statistical analyses and graphs were performed with Minitab version 19.0 (State College, Pennsylvania, USA) or using R (version 4.1.2) and RStudio (version 1.4.1717) software. All two-tailed *P*<0.05 were considered statistically significant.

## 3. Results

**Figure 1:**
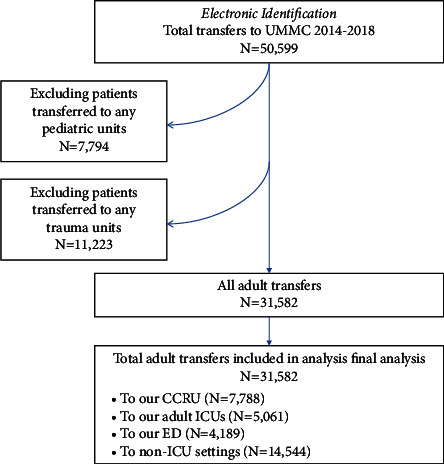
Flow diagram mapping patients included in the final analysis. Abbreviations: CCRU, critical care resuscitation unit; ED, emergency department; ICU, intensive care unit; UMMC, University of Maryland Medical Center.

**Figure 2:**
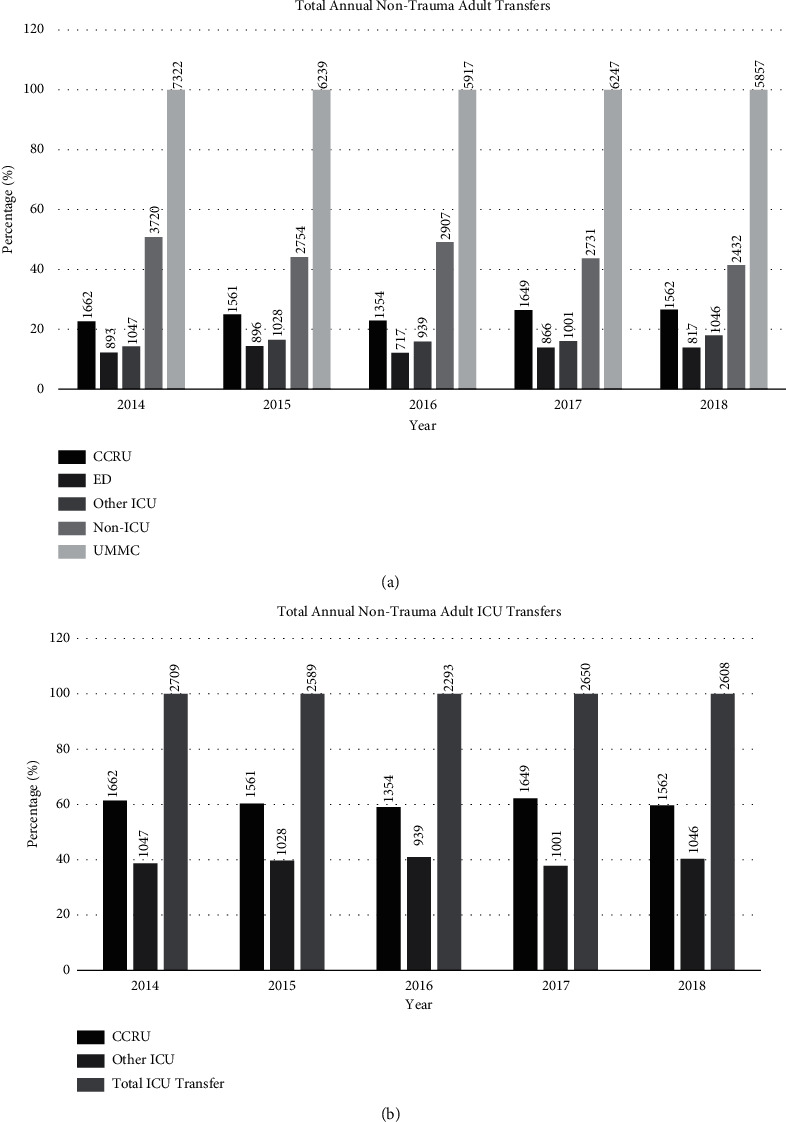
(a) Total annual non-trauma adult transfers from other hospitals to our academic medical center. The *y*-axis represents the number of patients as a percentage of the total adult transfers to our medical center, which is set as 100%. The *x*-axis shows the name of each unit per year. The number on each bar represents the number of transfers. Abbreviations: CCRU, critical care resuscitation unit; ED, emergency department; ICU, intensive care unit; UMMC, University of Maryland Medical Center. (b). Total annual non-trauma adult transfers from other hospitals to any one of our academic medical center's adult ICUs. The *y*-axis represents the number of patients as a percentage of the total adult transfers to any ICU at our medical center, which is set as 100%. The *x*-axis shows the name of each unit per year. The number on each bar represents the number of transfers. Abbreviations: CCRU, critical care resuscitation unit; ED, emergency department; ICU, intensive care unit; UMMC, University of Maryland Medical Center.

From 2014 to 2018, a total of 50,599 patients were transferred to our medical center (Figure 1). Of these patients, 7,794 were excluded as pediatric transfers and 11,223 transfers were excluded as trauma transfers. From a total of 31,582 non-trauma adult transfers, the CCRU received 7,788 (25%) patients during this period, the adult ED received 4,189 (13%) patients, all other adult ICUs at our center received 5,061 (16%) patients, and 14,544 (46%) patients went to non-ICU destinations (Figure 2(a)). The CCRU received the second most annual transfers, only behind transfers to all non-ICU inpatient units such as intermediate care units or surgical or medical wards (Figure 2(b)).

Demographic information for all adult transfers and transfers to the ED, ICUs, and CCRU are presented in Table 1. Patients from the adult ED were younger than those transferred to the ICU or CCRU (mean age, total/CCRU/ICU/ED: 55/57/59/47 years). Patients from the ED were less likely to arrive via air transport compared to ground transport. Ground transport was the most commonly used mode of transportation (41%), with only 5% of patients transferred by air. Community hospitals referred more patients to UMMC than teaching hospitals (64% community vs. 31% teaching), and most transfers were admitted on weekdays (23,549, 75%). When compared to other ICUs or the ED, the CCRU had the most weekend (1,957, 25%) and evening admissions (3,076, 39%).

Cardiology was the highest volume admitting service (4,566, 15%) and most of these admissions went directly to an ICU (1,296, 26%) or to another floor (3,133, 21%) rather than CCRU (137, 2%) or ED (0, 0%). The acute care emergency surgery service was the most common admitting service for patients transferred to the ED (22, 1%), whereas the most common accepting service for ICU transfer was the pulmonary and critical care medicine service (1,478, 29%), which is the designated term for our medical ICU (MICU). For the CCRU, the most common accepting services were cardiac surgery (1212, 16%), neurosurgery (858, 11%), and the acute care emergency surgery service (813, 10%).

**Figure 3:**
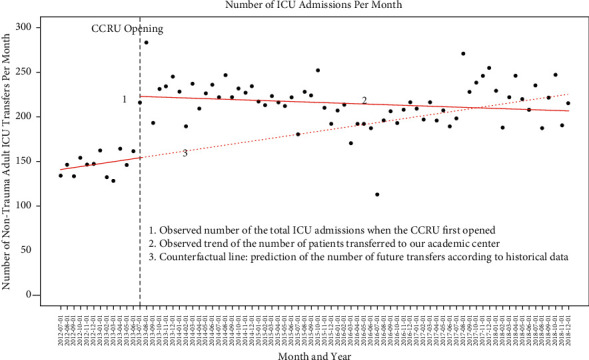
Interrupted time series (ITS) demonstrating the trend of non-trauma adult patients who were transferred from other hospitals to any adult intensive care unit (ICU) at our academic medical center, University of Maryland Medical Center (UMMC). The CCRU was opened in July 2013. Abbreviations: CCRU, critical care resuscitation unit; ICU, intensive care unit; UMMC, University of Maryland Medical Center.

Figure 3 is an ITS analysis demonstrating the trend of non-trauma adult patients who were transferred from referring hospitals to any ICU, including the CCRU, at our academic medical center from July 1, 2012 to December 31, 2018. Immediately after the opening of the CCRU in July 2013, a significant increase in the number of ICU transfers was observed (coefficient 138, *P* < 0.001) (Point 1, Figure 3). Throughout the time-series analysis, the period after the opening of the CCRU was associated with significantly more ICU transfers per month (level = 72, *P* < 0.001). There was an average of 72 more ICU transfers per month from when the CCRU opened until the end of our study period when compared to the historical number of ICU admissions. However, the trend of total ICU admissions after the CCRU opening was not significantly higher than the trend from historical data (trend = −1.4, *P*=0.49).

The median (interquartile range [IQR]) time interval from transfer request to bed assignment for our entire hospital was 121 [40–411] minutes. Our ED (23 [11–56] minutes) was associated with the shortest median time interval from transfer request to bed assignment, while the time intervals for CCRU patients were significantly less (46 [22–139] minutes) when compared with other traditional ICU (156 [65–422], minutes, *P* < 0.001) or other non-ICU inpatient units (259 [65–1027] minutes, *P* < 0.001) (Table 1).

**Figure 4:**
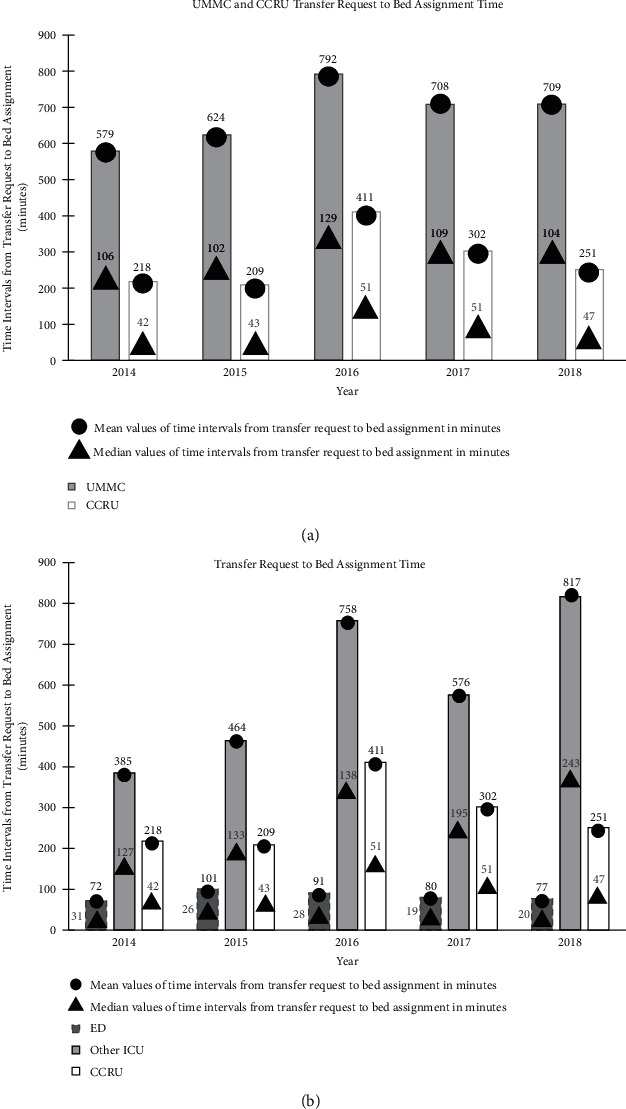
(a) Comparison of time intervals from transfer request to bed assignment for the critical care resuscitation unit (CCRU) and overall time intervals for transfer to our academic medical center (UMMC). Median of time intervals for UMMC and CCRU patients were analyzed using Mann–Whitney *U* tests with all *P*-values<0.001. Abbreviations: CCRU, critical care resuscitation unit; UMMC, University of Maryland Medical Center. (b). Comparison of time intervals from transfer request to each unit's bed assignment for patients who were transferred from other hospitals to our academic medical center. Median time intervals for CCRU vs. other ICU and CCRU vs. ED transfer were analyzed using Mann–Whitney *U* tests with all *P*-values<0.001. Abbreviations: CCRU, critical care resuscitation unit; ED, emergency department; ICU, intensive care unit; UMMC, University of Maryland Medical Center.

**Figure 5:**
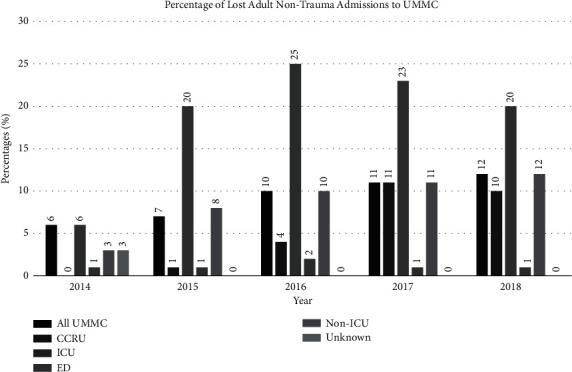
Lost admissions to UMMC units over the 5-year study period. Abbreviations: CCRU, critical care resuscitation unit; ED, emergency department; ICU, intensive care unit; UMMC, University of Maryland Medical Center.

Compared to all hospital admissions, the CCRU had a significantly faster time interval from transfer request to bed assignment across the study period (Figure 4(a)). The ED had the fastest yearly time from transfer request to bed assignment across each year of the study period, while the CCRU had a significantly faster time per year compared to other ICUs (Figure 4(b)). The volume of lost admissions (Figure 5) showed a trend that increased over time but was stabilized at approximately 10% of the total transfer request.

## 4. Discussion

### 4.1. Access to Definitive Care and Interventions

Our data demonstrate that over the 5-year period, the CCRU contributes significantly to overall hospital admissions. This increased transfer capability allows access to specialty care for patients in our region while decreasing transfer times compared to other traditional ICUs.

Historically, delayed transfer to ICU beds has increased mortality, and previous studies demonstrated a limitation to access to subspecialty services offered at our tertiary care facility due to lack of bed availability [11]. A previous study involving patients who were transferred to our academic center in a year prior to the CCRU opening and after the establishment of the CCRU demonstrated that transferring to the CCRU was associated with improved patient outcomes, likely due to earlier access to definitive care [7]. ED-based ICUs (ED-ICUs) have also previously demonstrated improved patient outcomes likely due to a similar mechanism [12]. Given additional access to care, we would expect to see an improvement in morbidity and mortality; however, this was not specifically investigated in this study. Future studies are necessary to confirm whether the improved outcomes are sustained over time, as both previous studies only utilized 1 year of data after the respective resuscitation units were operational [7, 12].

### 4.2. Impact on Emergency Department Crowding

ED crowding is associated with worse patient outcomes, increased length of stay, and increased overall costs [13–15]. ED-ICUs improve patient outcomes while also lowering hospital length of stay and decreasing ICU admissions [12, 16, 17]. The ED-ICU reduces time from arrival to the ED to ICU care and allows for critically ill patients in the ED to receive attention from ED physicians and ED nursing staff. These specialized resuscitation units are one facet of addressing ED crowding [18].

It is likely that the CCRU contributed to lowering the burden of ED crowding, both from the referring hospital and in the receiving ED at our medical center; however, we did not specifically quantify this effect. Our 6-bed CCRU accommodated 7,788 or approximately 25% of transfers of the entire 800-bed hospital over the study period. Presumably, many of these patients would otherwise need to go to our medical center's ED, especially in the most emergent cases. Patient transfer to the ED is complicated by the number of hours that our ED is full and the patients who are transferred to our medical center's ED may have prolonged ED boarding time, while not having the same ICU-level ratio of nurses-to-patients or intensivists' clinical expertise. Furthermore, the data demonstrated that the overall number of “lost admissions,” from other hospitals' EDs or inpatient units to our quaternary medical center remained stabilized at approximately 10%, which was significantly reduced from the 25% during the year prior to the opening of the CCRU [6].

### 4.3. Hospital Revenue and Financial Sustainability

The CCRU provides access to specialty services for patients while allowing the continuation of previously scheduled surgical cases. Prior to the establishment of the CCRU, any urgent or emergent cardiac surgery or neurosurgery transfers would need to go directly to the CSICU or the NCCU. Given the limited bed availability, this would lead to the cancelling of previously planned cases that would require a postoperative ICU. The coronavirus disease 2019 (COVID-19) pandemic has demonstrated that cancelling scheduled and elective surgical cases results in the loss of revenue for hospitals [19]. Hospitals that cancel surgical cases due to strained ICU capacity have also been shown to decrease hospital revenue [20]. The multispecialty expertise of the CCRU allows patients with a wide range of disease processes to be transferred and to receive prompt resuscitation while also allowing destination units to continue to conduct scheduled surgical cases that would have otherwise been postponed or cancelled if another admission occupied the patient's bed in the ICU. Further investigation into the specific implications for surgical revenue is needed.

The United States has seen an increase in the number of ICU beds in recent history [21]. However, increasing the number of traditional ICU beds can result in increased hospital fixed costs while creating waste in the system when these beds are not occupied [22]. There have been questions regarding the cost-effectiveness of shorter stay resuscitation/ICU units as a potential cost-saving alternative [23]. One study examined creating a shorter stay unit for intermediate risk patients who may not require long-term ICU management. These beds provide ICU capability but less cost to the system overall [24]. The CCRU provides a similar short stay model where patients may be triaged to their destination ICU or downgraded based on clinical improvement. The CCRU allows for an increase in ICU admissions without having to staff and maintain additional traditional ICU beds, and thus we would expect a cost benefit to the health system; however, this was not specifically studied.

### 4.4. In-Hospital Patient Safety

In addition to facilitating the interhospital patient transfer, the CCRU provides the capability to immediately admit patients from our medical center's regular wards or step-down units who clinically deteriorate when other traditional ICUs at our hospital are full. Due to limited ICU bed availability, there is a risk for delayed ICU transfer after a rapid response or cardiac arrests in non-ICUs. Delay in ICU transfer from within the same hospital increases mortality [25]. The presence of a specialized resuscitation unit that can rapidly admit unstable patients throughout the hospital may improve patient outcomes.

### 4.5. Limitations

As this is a single-center study of a novel resuscitation unit, our findings may not be generalizable, as a different resuscitation unit may not function as a regional ICU. Further limiting our analysis was missing and unavailable data from the transferring hospitals. We utilized the number of patients who were transferred in the year prior to the CCRU opening for our ITS, but there were missing data that prevented us from being able to use this cohort to establish a pre-CCRU control group. Our 5-year analysis naturally captured periods where the ICU would be at full capacity and periods where the ICU would have available beds. Although ICU congestion was one of the major reasons for the establishment of the CCRU, we did not fully consider reasons for ICU congestion or other factors that could overall increase the transfer volume to our hospital. However, a major strength of our study is that our analysis occurred over the 5-year period with an ITS to account for seasonal variabilities that could have occurred. Mortality and cost were also not specifically examined as these variables were not part of the MEC transfer center's clinical data and were beyond the scope of this study. However, previous studies suggested that surgical patients who were transferred from other hospitals were associated with higher costs and poorer outcomes; therefore, our CCRU patients would be expected to have worse outcomes than patients who initially presented to our ED or other non-ICUs [26, 27]. We also did not assess the time intervals from transfer request to arrival at our medical center, as this variable may not truly reflect our medical center's operations and is confounded by the sending hospital and the referring physician's transfer practices once a bed at UMMC is assigned.

## 5. Conclusions

The CCRU is a unique model benefitting both patients and the health system by facilitating necessary transfers while reducing “lost admissions.” This may improve patient outcomes and hospital revenue. Our study demonstrated that the CCRU model is sustainable over the 5-year period. Further research on patient outcomes and cost-effectiveness is needed.

## Figures and Tables

**Table 1:** Demographics and clinical features of patients from all UMMC units during the 5-year study period.

Variables	All UMMC^*∗*^	UMMC CCRU	UMMC ICU^*∗∗*^	UMMC ED	UMMC other inpatient units	*P* ^†^
Total patients, *N*, (%)	31582 (100)	7788 (25)	5061 (16)	4189 (13)	14544 (46)	NA

Age (years), mean (SD)	55 (17)	57 (16)	59 (16)	47 (18)	56 (18)	**<0.001**, **<0.001**, **<0.001**

Type of transport, *N* (%)^^#^						
Air	1500 (5)	890 (11)	304 (6)	25 (1)	281 (4)	**<0.001**, **<0.001**, **<0.001** **<0.001**, **<0.001**, **<0.001**
Ground	13024 (41)	2887 (37)	2213 (44)	1762 (42)	6162 (42)	
Unknown	17058 (54)	4011 (52)	2544 (50)	2402 (57)	8101 (56)	

Ground distance (km), median [IQR]	42 [14–77]	50 [15–81]	47 [13–78]	22 [5–54]	50 [22–94]	**0.001**, **<0.001**, 0.82

Number of hospital beds, median [IQR]	183 [131–268]	200 [137–272]	182 [137–272]	170 [109–257]	178 [139–272]	**0.028**, **<0.001**, **0.001**

Type of referring hospital, *N* (%)^^^						
Teaching	9719 (31)	2650 (34)	1710 (34)	1124 (27)	4235 (29)	0.71, 0.49, **<0.001** 0.60, **<0.001**, **<0.001**
Community	20305 (64)	5006 (64)	3276 (65)	2059 (49)	9964 (69)	
Other/unknown	1558 (5)	132 (2)	75 (1)	1006 (24)	345 (2)	

Transfer request to bed assignment (minute), median [IQR]	121 [40–411]	46 [22–139]	156 [65–422]	23 [11–56]	259 [65–1027]	**<0.001**, **<0.001**, **<0.001**

Admission day of the week, *N* (%)						
Weekday (Monday–Friday)	23549 (75)	5831 (75)	3732 (74)	2861 (68)	11125 (76)	0.15, **<0.001**, **0.007**
Weekend (Saturday–Sunday)	8033 (25)	1957 (25)	1329 (26)	1328 (32)	3419 (24)	

Admission time of the day, *N* (%)						
Day time (07 : 00–19 : 00)	19887 (63)	4712 (61)	3197 (63)	2423 (58)	9555 (66)	**0.002**, **0.005**, **<0.001**
Evening time (19 : 01–06 : 59)	11695 (37)	3076 (39)	1864 (37)	1766 (42)	4989 (34)	

Accepting service, *N* (%)^^^						
Emergency general surgery	1382 (4)	813 (10)	9 (0)	22 (1)	538 (4)	**<0.001**, NA, **<0.001** **<0.001**, **<0.001**, **<0.001**
Cardiac surgery	3089 (10)	1212 (16)	294 (6)	1 (0)	1582 (11)	
Cardiology	4566 (15)	137 (2)	1296 (26)	0 (0)	3133 (21)	
Neurology	1962 (6)	770 (10)	702 (14)	1 (0)	489 (3)	
Neurosurgery	1836 (6)	858 (11)	731 (14)	1 (0)	246 (2)	
Oncology	1296 (4)	103 (1)	2 (0)	0 (0)	1191 (8)	
Pulmonary and critical care	1883 (6)	391 (5)	1478 (29)	0 (0)	14 (0)	
Thoracic surgery	410 (1)	163 (2)	3 (0)	0 (0)	244 (2)	
Transplant	1662 (5)	163 (2)	12 (0)	1 (0)	1486 (10)	
Vascular surgery	1099 (4)	663 (9)	7 (0)	1 (0)	428 (3)	
Other accepting services	12397 (39)	2515 (32)	527 (11)	4162 (99)	5193 (36)	

Abbreviations: CCRU, critical care resuscitation unit; ED, emergency department; ICU, intensive care unit; IQR, interquartile range; min, minutes; NA, not applicable; km, kilometers; SD, standard deviation; UMMC, University of Maryland Medical Center. ^*∗*^All UMMC units do not include pediatric or trauma patients. ^*∗∗*^ICU patients are separate from CCRU. ^#^Transport type was only indicated for patients transferred after July 2016. ^^^Indicates that the top group of *P*-values was calculated excluding the unknown or other/unknown group and that the bottom group of *P*-values was calculated with the unknown or other/unknown groups with either Pearson's chi-squared test or Fisher's exact test as appropriate. ^†^Bold cells indicate statistically significant findings (*P* < 0.05). *P*-values are written as *P*_1_, *P*_2_, *P*_3_ where: *P*_1_ = *CCRU* vs. ICU *P*_2_ = *CCRU* vs. ED *P*_3_ = *CCRU* vs. other inpatient units.

## Data Availability

In accordance with the IRB agreement with the University of Maryland Baltimore, the data presented in this manuscript may not be shared or distributed.
